# Safe and sensible preprocessing and baseline correction of pupil-size data

**DOI:** 10.3758/s13428-017-1007-2

**Published:** 2018-01-12

**Authors:** Sebastiaan Mathôt, Jasper Fabius, Elle Van Heusden, Stefan Van der Stigchel

**Affiliations:** 1 Department of Psychology, Heymans Institute, University of Groningen, Groningen, The Netherlands; 2 Aix-Marseille University, CNRS, LPC UMR 7290, Marseille, France; 3 Department of Experimental Psychology, Helmholtz Institute, Utrecht University, Utrecht, Netherlands

**Keywords:** Pupillometry, Pupil size, Baseline correction, Research methods

## Abstract

Measurement of pupil size (pupillometry) has recently gained renewed interest from psychologists, but there is little agreement on how pupil-size data is best analyzed. Here we focus on one aspect of pupillometric analyses: baseline correction, i.e., analyzing changes in pupil size relative to a baseline period. Baseline correction is useful in experiments that investigate the effect of some experimental manipulation on pupil size. In such experiments, baseline correction improves statistical power by taking into account random fluctuations in pupil size over time. However, we show that baseline correction can also distort data if unrealistically small pupil sizes are recorded during the baseline period, which can easily occur due to eye blinks, data loss, or other distortions. Divisive baseline correction (corrected pupil size = pupil size/baseline) is affected more strongly by such distortions than subtractive baseline correction (corrected pupil size = pupil size − baseline). We discuss the role of baseline correction as a part of preprocessing of pupillometric data, and make five recommendations: (1) before baseline correction, perform data preprocessing to mark missing and invalid data, but assume that some distortions will remain in the data; (2) use subtractive baseline correction; (3) visually compare your corrected and uncorrected data; (4) be wary of pupil-size effects that emerge faster than the latency of the pupillary response allows (within ±220 ms after the manipulation that induces the effect); and (5) remove trials on which baseline pupil size is unrealistically small (indicative of blinks and other distortions).

Pupil size is a continuous signal: a series of values that indicate how pupil size changes over time. In this sense, pupil-size data are similar to physiological measures, such as electroencephalography (EEG), which measures electrical brain activity over time, and it is even more similar to skin conductance, which (like pupil size) fluctuates slowly and correlates with arousal (Bradley, Miccoli, Escrig, & Lang, 2008). Pupil size is different from most behavioral measures, such as response times, that generally provide only a single value for each trial of the experiment.

Psychologists are often interested in how pupil size is affected by some experimental manipulation (reviewed in Beatty & Lucero-Wagoner, 2000; Mathôt & Van der Stigchel, 2015). To give a classic example, Kahneman and Beatty (1966) asked participants to remember a varying number (three to seven) of digits. They found that participants’ pupils dilated (i.e., became larger) when the participants remembered seven digits, compared to when they remembered only three; that is, memory load caused the pupil to dilate (become bigger).

As was common for pupil-size studies of the time, Kahneman and Beatty (1966) expressed their results in millimeters of pupil diameter; that is, they used absolute pupil-size values. But expressing pupil size in absolute values has a disadvantage: It is affected by slow, random fluctuations of pupil size. These fluctuations are a source of noise that reduce statistical power and make it more difficult to detect the effects of interest (in the case of Kahneman & Beatty, 1966, the effect of memory load). To deal with these fluctuations, researchers often look at the *difference* in pupil size compared to a baseline period, which is typically the start of the trial. By looking at pupil-size changes, rather than absolute pupil sizes, differences in pupil size that already existed before the trial are taken into account, are no longer a source of noise, and no longer reduce statistical power. This is baseline correction.

There are two main ways to apply baseline correction: *divisive*, in which pupil size is converted to a proportional difference from baseline pupil size (corrected pupil size = pupil size/baseline), and *subtractive*, in which pupil size is converted to an absolute difference from baseline pupil size (corrected pupil size = pupil size − baseline). There are variations of these approaches, such as using percentage rather than proportion change, or converting absolute differences from baseline pupil size to z-scores; but these are all minor variations of these two general approaches. Here we will therefore focus on the difference between divisive and subtractive baseline correction.

There are several reasons why researchers may choose either divisive or subtractive baseline correction. (Although such reasons are rarely provided.) Divisive baseline correction is attractive because it provides an intuitive measure: proportion change. If a paper states that an eye movement caused a 10 % pupillary constriction (Mathôt, Melmi, & Castet, 2015d), you can easily judge the size of this effect: substantial but not enormous. In contrast, if a paper states that a manipulation caused a 0.02-mm diameter change (Bombeke, Duthoo, Mueller, Hopf, & Boehler, 2016), you need a moment to remember (or look up) that human pupils are 2–8 mm in diameter, and that a 0.02-mm effect is therefore tiny. This is, in our view, less intuitive. And if the eye tracker reports pupil size in arbitrary units, typically based on a pixel count of the camera image, then absolute pupil-size differences become even harder to interpret. (In addition, even for eye trackers that report pupil size in seemingly absolute units [mm], measurements may not be entirely invariant to factors such as the distance between the participant and the camera, and may therefore be partly arbitrary as well.) However, despite these disadvantages, subtractive baseline correction may be the natural choice for some researchers because it is the standard approach in EEG research (e.g., Gross et al., 2013; Woodman, 2010).

In pupillometry, there is no established standard for applying baseline correction. Based on our experience, most researchers now apply some form of baseline correction (but see, e.g., Gamlin et al., 2007), and variations of subtractive baseline correction (Binda, Pereverzeva, & Murray, 2013; Hupé, Lamirel, & Lorenceau, 2009; Jainta, Vernet, Yang, & Kapoula, 2011; Knapen et al., 2016; Koelewijn, Zekveld, Festen, & Kramer, 2012; e.g. Laeng & Sulutvedt, 2014; Murphy, Moort, & Nieuwenhuis, 2016; Porter, Troscianko, & Gilchrist, 2007; Privitera, Renninger, Carney, Klein, & Aguilar, 2010) seem somewhat more common than variations of divisive baseline correction (Bonmati-Carrion et al., 2016; Herbst, Sander, Milea, Lund-Andersen, & Kawasaki, 2011; Mathôt, van der Linden, Grainger, & Vitu, 2013; H. Wilhelm, Lüdtke, & Wilhelm, 1998). (One paper is listed per research group. This list is anecdotal, and not a comprehensive review.)

As far as we know, no-one has systematically studied baseline correction of pupil-size data. However, baseline correction has been studied in the context of EEG/MEG data, as shown by a recent debate about whether or not baseline correction of EEG/MEG data should be abandoned in favor of high-pass filtering (Maess, Schröger, & Widmann, 2016; Tanner, Morgan-Short, & Luck, 2015; Tanner, Norton, Morgan-Short, & Luck, 2016). However, pupil-size data are different from EEG/ MEG data. For example, although in the absence of direct external stimulation the size of the pupil fluctuates in cycles of 1–2 s (Mathôt, Siebold, Donk, & Vitu, 2015a; Reimer et al., 2014), it does not show the slow systematic drift shown by EEG/MEG voltages (Tanner et al., 2016).

Our aim is therefore to study baseline correction specifically for pupil-size data. We will use real and simulated data to see how robust subtractive and divisive baseline corrections are to noise, and how they affect statistical power.

In addition to performing baseline correction, researchers generally preprocess pupil-size data in several ways, such as smoothing or interpolation of missing data. Therefore, although baseline correction is our main focus, we will start with a brief, general overview of preprocessing of pupil-size data. However, for our analyses we will not apply any other preprocessing steps, because we feel that baseline correction should be safe (i.e., not increase the probability of false alarms and misses) and sensible on its own. We will end by making several recommendations for preprocessing and baseline correction of pupil-size data.

## A preprocessing primer

Baseline correction is part of what researchers often refer to as *preprocessing*: cleaning the raw data (i.e., as recorded by the eye tracker) from as many undesirable features as possible. Of course, what constitutes an undesirable feature depends on the context. For example, eye blinks are often considered undesirable in pupillometry, because you cannot measure pupil size while the eyes are closed, and also because blinks are followed by a prolonged constriction of the pupil (Knapen et al., 2016). But in a different context, for example when using blink rate as a measure of fatigue (Stern, Boyer, & Schroeder, 1994), eye blinks can also be the measure of interest. In other words, preprocessing is performed differently in different situations.

For pupillometry research, we recommend the following preprocessing steps. Baseline correction—the focus of this paper—is generally performed last.

### Dealing with missing data

Missing data most often occurs when a video-based eye tracker fails to extract the pupil from the camera image. Most eye trackers will then report a pupil size of 0, or indicate in some other way that data are missing. The first step in dealing with missing data is therefore to find out how the eye tracker reports missing data (e.g., as 0s), and then to ignore these values, for example by treating them as *nan* (not a number) values during the analysis; nan values are offered by most modern programming languages, and are a standard way to silently ignore missing data.

Whether missing data deserve special treatment is a matter of opinion. Some researchers prefer to interpolate missing data (e.g., Knapen et al., 2016), similar to eye-blink reconstruction, as discussed below. Other researchers prefer to exclude trials with too much missing data (e.g., Koelewijn et al., 2012). In our view, missing data need no special treatment because what is not there does not affect the results anyway—as long as care is taken that missing data are not interpreted as real 0 pupil-size measurements, and as long as the proportion of missing data is independent of pupil size (i.e., data for large pupils is equally likely to be missing as for small pupils) and experimental conditions (i.e., data in one condition is equally likely to be missing as for another condition).

### Dealing with eye blinks and other unrealistic pupil size values

A bigger problem occurs when pupil-size values are reported incorrectly but not marked as missing data. The size of the pupil changes at most by a factor of around 4, when expressed as pupil diameter, or around 16, when expressed as pupil surface (McDougal & Gamlin, 2008). If pupil size, as measured, changes more than this, this means that something distorted the recording. By far the most common source of distortion is eye blinks; eye blinks are characterized by a rapid decrease in pupil size, followed by a period of missing data, followed by a rapid increase in pupil size. The period of missing data can be treated (or not) as discussed above; but the preceding and following distortions should be corrected, because they can strongly affect pupil size, even when averaged across many trials.

One way to deal with eye blinks, and our preferred method, is to use cubic-spline interpolation. This method is described in more detail elsewhere (Mathôt, 2013), but in a nutshell works as follows: Four points (A, B, C, and D) are placed around the on- and offset of the blink. Point B is placed slightly before the onset of the blink; point C is placed slightly after the onset of the blink. Point A is then placed before point B; point D is placed after point C. Points are equally spaced, such that the distances between A and B, B and C, etc. are constant. Finally, a smooth line is drawn through all four points, replacing the missing and distorted data between B and C.

### Dealing with position artifacts

Imagine that a participant looks directly at the lens of a video-based eye tracker. The pupil is then recorded as a near-perfect circle. Now imagine that the participant makes an eye movement to the right, thus causing the eye ball to rotate, changing the angle from which the eye tracker records the pupil, and causing the horizontal diameter of the pupil (as recorded) to decrease. In other words, pupil size as recorded by the eye tracker decreases, even though pupil size really did not change. Most commonly used eye trackers, such as the EyeLink (SR Research, Missisauga, Ontario, Canada), cannot distinguish such artifactual pupil-size changes due to eye movements from real pupil-size changes. Eye trackers that work with a physical model of the eye, such as Pupil Labs (Pupil Labs, Berlin, Germany), in theory could distinguish artifactual from real pupil-size changes, as described below.

Importantly, artifactual pupil-size changes can be larger than the real pupil-size changes that are induced by many psychological manipulations. For example, Fig. 5 from Mathôt, Melmi, and Castet (2015d) shows pupil size during eye movements in four directions (leftward, rightward, upward, and downward). In this particular set-up, a downward eye movement movement caused an artifactual increase in pupil size of 10 %. This is comparable to the largest (real) change in pupil size that Kahneman and Beatty (1966) observed in a working-memory task.

There are three main ways to take position artifacts in pupil-size data into account: comparing conditions that are matched in terms of eye position (e.g., Gagl, Hawelka, & Hutzler, 2011); using data-driven correction, which uses linear regression to remove position artifacts from pupil-size data (e.g., Brisson et al., 2013); and model-driven correction, which uses knowledge about the relative positions of the camera, eyes, and eye tracker (e.g., Gagl et al., 2011; Hayes & Petrov, 2016).

When comparing conditions that are matched on eye position, the eyes may still move, as long as they do so in the same way in all conditions. For example, Gagl et al. (2011) compared pupil size between a condition in which participants read real sentences, and a condition in which participants read z-strings (i.e., strings where all characters of the real text were replaced by z’s). Because eye movements did not differ between these two conditions, any difference in pupil size is real and due to the difference between conditions (in this case due to the cognitive processes involved in reading). Whenever you are not interested in absolute pupil-size measurements, and whenever it is practically feasible to match eye position between conditions, this is unequivocally the best way to take position artifacts into account—because it does not rely on any assumptions.

Data-driven correction involves a calibration procedure during which participants look at points on different parts of the screen (e.g., Brisson et al., 2013). A multiple linear regression is then performed to predict pupil size from horizontal (X) and vertical (Y) pupil size. Using this regression, position artifacts could then be removed from pupil size during the rest of the experiment. This procedure assumes that, in reality, pupil size does not depend on eye position, and any measured relationship is therefore artifactual.

Unfortunately, this procedure does not work for the simple reason that pupil size may actually (and usually does) change as a function of eye position. For example, if participants need to look up uncomfortably to foveate the top of the screen, the mental and physical effort involved may cause their pupils to dilate. When performing a correction as described above, this real pupil-size change would be corrected as though it were artifactual. Even worse, artifactual pupil-size changes happen immediately when the eyes move, whereas real pupil-size changes occur slowly. Therefore, they have to be treated differently; if not, pupil size may seem completely free of position artifacts just before each eye movement, but catastrophically affected by position artifacts immediately after each eye movement. We have seen this in our data, and decided to leave position artifacts uncorrected for exactly this reason (Mathôt et al., 2015d).

Model-based correction requires knowledge of the geometry of the eyes and the recording set-up: the position of the eyes relative to the display and the eye tracker (e.g., Gagl et al., 2011; Hayes & Petrov, 2016), and, for even more accurate correction, the thickness of the iris (e.g., Gagl et al., 2011) and the focusing power of the cornea and lens, which affect the appearance of the pupil, especially when viewed off-axis (e.g., Mathur, Gehrmann, & Atchison, 2013). Once you have constructed a physical model based on this information, position artifacts can be isolated and removed from pupil-size measurements. Studies using an artificial pupil of fixed size (so that true pupil size is known) have shown that model-based correction is highly effective (Gagl et al., 2011; Hayes & Petrov, 2016). Crucially, and unlike data-driven correction, model-based correction does not inappropriately “correct” for real pupil size changes that may accompany changes in gaze position.

To summarize, the size of the pupil changes as a function of eye position, and this is in part an artifact due to the angle from which the eye tracker records the pupil. Ideally, pupil size is compared between conditions that are matched on eye position, in which case pupil-size differences between conditions are not confounded by position artifacts. When such matching is not feasible, the best way to remove position artifacts from pupil-size data is using a model-based correction that takes into account the geometry of the eye and the recording set-up (e.g., Gagl et al., 2011; Hayes & Petrov, 2016).

### Hope for the best, prepare for the worst

Data preprocessing is useful, improves statistical power (the probability of detecting true effects), reduces the probability of spurious results, and makes it substantially easier to analyze and interpret results. However, eye-movement data are messy, and every form of preprocessing is guaranteed to fail occasionally. For example, blink reconstruction often fails when the eyes close only partly (is that even a blink?) or when due to noise the blink is not detected.

Therefore, results should not crucially depend on whether the data still contain artifacts. Similarly, one preprocessing step should not crucially depend on whether the previous preprocessing step was perfect. For this reason, in this paper we focus on baseline correction in isolation, as if no preprocessing steps are performed beforehand. However, in real-life situations, baseline correction generally is—and should be—accompanied by others forms of preprocessing.

## Simulated data

We first investigate the effect of divisive and subtractive baseline correction in simulated data. The advantage of simulated data over real data is that it allows us to control noise and distortion, and therefore to see how robust baseline correction is to imperfections of the kind that also occur in real data. In addition, simulated data allow us to simulate two experimental conditions that differ in pupil size by a known amount, and therefore to see how baseline correction affects the power to detect this difference.

### Data generation

We started with a single real 3-s recording of a pupillary response to light, recorded at 1,000 Hz with an EyeLink 1000 (SR Research). This recording did not contain blinks or recording artifacts, but did contain the slight noise that is typical of pupil-size recordings. Pupil size was measured in arbitrary units as recorded by the eye tracker, ranging from roughly 1,600 to 4,200.

**Fig. 1:**
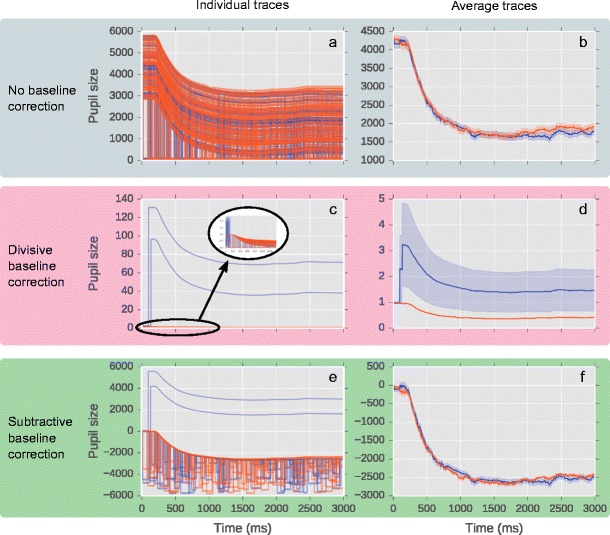
The effects of divisive and subtractive baseline correction in a simulated dataset. (**a**, **b**) No baseline correction. Y-axis reflects pupil size in arbitrary units. (**c**, **d**) Divisive baseline correction. Y-axis reflects proportional pupil-size change relative to baseline period. (**e**, **f**) Subtractive baseline correction. Y-axis reflects difference in pupil-size from baseline period in arbitrary units. Individual pupil traces: (**a**, **c**, **e**). Average pupil traces: (**b**, **d**, **f**)

Based on this single recording, 200 trials were generated. To each trial, a constant value, randomly chosen for each trial, between -1,000 and 2,000 was added to each sample. In Fig. 1b, this is visible as a random shift of each trace up or down the Y-axis. In addition, one simulated eye blink was added to each trial. Eye blinks were modeled as a period of 10 ms during which pupil size linearly decreased to 0, followed by 50–150 ms (randomly varied) during which pupil size randomly fluctuated between 0 and 100, followed by a period of 10 ms during which pupil size linearly increased back to its normal value. This resembles real eye blinks as they are recorded by video-based eye trackers (e.g., Mathôt, 2013).

To simulate two conditions that differed in pupil size, we added a series of values that linearly increased from 0 (at the start of the trial) to 200 (at the end of the trial) to half the trials (i.e., the same slight increase from 0 to 200 was applied to half the trials). These trials are the Red condition; the other trials are the Blue condition. As shown in Fig. 1a, pupil size is slightly larger in the Red condition than in the Blue condition, and this effect increases over time.

Crucially, in the Blue condition there were two trials in which a blink started at the first sample, and therefore affected the baseline period. In none of the other trials did the baseline period contain a blink. By making the two conditions equally “noisy” (i.e., with exactly one blink per trial) but having two trials in the Blue condition in which these blinks occurred during the baseline, we can show how only a few trials with artifacts during the baseline can dramatically affect the overall results. Crucially, this can easily happen by chance, and even thorough data preprocessing does not guarantee that it will not.

### Divisive baseline correction

First, median pupil size during the first ten samples (corresponding to 10 ms) was taken as baseline pupil size. Next, all pupil sizes were divided by this baseline pupil size. This was done separately for each trial.

(The length of the baseline period varies strongly from study to study. Some authors prefer long baseline periods of up to 1,000 ms (e.g., Laeng & Sulutvedt, 2014), which have the disadvantage of being susceptible to pupil-size fluctuations during the baseline period. Other authors, including we (e.g., Mathôt et al., 2015d), prefer short baseline periods, which have the disadvantage of being susceptible to recording noise. But the problems that we highlight in this paper apply to long and short baseline periods alike.)

The results of divisive baseline correction are shown in Fig. 1c,d. In the two Blue trials in which there was a blink during the baseline period, baseline pupil size was very small; consequently, baseline-corrected pupil size was very large. These two trials are clearly visible in Fig. 1c as unrealistic baseline-corrected pupil sizes ranging from 40 to 130 (as a proportion of baseline), whereas in this dataset realistic baseline-corrected pupil sizes tend to range from 0.3 to 1 (see zoom panel in Fig. 1c). Pupil sizes on these two trials are so strongly distorted that they even affect the overall results: As shown in Fig. 1d, the overall results suggest that the pupil is largest in the Blue condition, whereas we had simulated an effect in the opposite direction.

### Subtractive baseline correction

Subtractive baseline correction was identical to divisive baseline correction, except that baseline pupil size was subtracted from all pupil sizes.

The results of subtractive baseline correction are shown in Fig. 1e,f. Again, the two Blue trials with a blink during the baseline period are clearly visible in Fig. 1e. However, their effect on the overall dataset is not as catastrophic as for divisive baseline correction.

### Statistical power

The results above show that the effects of blinks during the baseline period can be catastrophic, and much more so for divisive than subtractive baseline correction. However, it may be that divisive baseline correction nevertheless leads to the highest statistical power when there are no blinks during the baseline period (even though this is unlikely to happen in real data). To test this, we generated data as described above, while varying the following:

- Effect size (Red larger than Blue), from 50 (±2 %) to 500 (±20 %) in steps of 50
- Baseline correction: no correction, divisive, or subtractive
- Blinks during baseline in Blue condition: yes (two blinks) or no

We generated 10,000 datasets for each combination, giving a total of (10,000 × 10 × 3 × 2 =) 600,000 datasets. For each dataset, we conducted an independent-samples t-test to test for a difference in mean pupil size between the Red and Blue conditions during the last 50 samples. We considered three possible outcomes:

- Detection of a true effect: *p* < .05 and pupil size smallest in the Blue condition
- Detection of a spurious effect: *p* < .05 and pupil smallest in the Red condition
- No detection of an effect: *p* ≥ .05

**Fig. 2:**
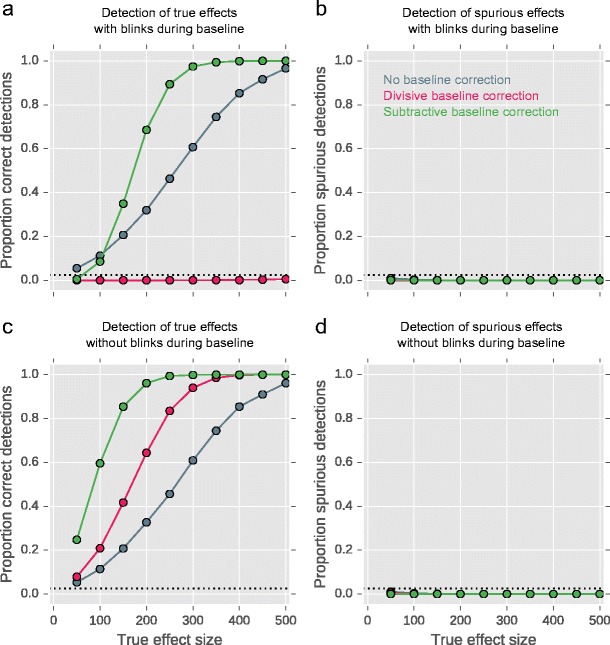
Proportion of detected real (**a**, **c**) and spurious (**b**, **d**) effects when applying subtractive baseline correction (green), divisive baseline correction (pink), or no baseline correction at all (gray). Data with different effect sizes (X-axis: 0*–*500) and with (**a**, **b**) or without (**c**, **d**) blinks during the baseline was generated

The proportion of datasets in which a true effect was detected is shown in Fig. 2a,c; the proportion on which a spurious effect was detected is shown in Fig. 2b,d. By chance (i.e., if there was no effect and no systematic distortion of the data) and given our two-sided *p* < .05 criterion, we would expect to find a .025 proportion of detections of true and spurious effects; this is indicated by the horizontal dotted lines.

First, consider the data with blinks in the baseline (Fig. 2a,b). With divisive baseline correction (pink), neither true nor spurious effects are detected (except for a handful of true effects for the highest effect sizes, but these are so few that they are hardly visible in the figure), because the blinks introduce so much variability in the signal that it is nearly impossible for any effect to be detected.

With subtractive baseline correction (green), true effects are often observed, and spurious effects are not. However, for weak effects, subtractive baseline correction is less sensitive than no baseline correction at all (gray). This is because, for weak effects, the blinks introduce so much variability that true effects cannot be detected; however, the variability is less than for divisive baseline correction, and for medium-to-large effects subtractive baseline correction is actually more sensitive than no baseline correction at all—despite blinks during the baseline.

Now consider the data without blinks in the baseline (Fig. 2b). An effect in the true direction is now detected in all cases, but there is a clear difference in sensitivity: subtractive baseline correction is most sensitive, followed by divisive baseline correction, in turn followed by no baseline correction at all. The three approaches do not differ markedly in the number of detected spurious effects.

## Real data

The simulated data highlights problems that can occur when applying baseline correction, especially divisive baseline correction, if there are blinks in the baseline period. However, you may wonder whether these problems actually occur in real data. To test this, we looked at the effects of baseline correction in one representative set of real data.

### Data

The data were collected for a different study, and consisted of 2,520 trials (across 15 participants) in two conditions, here labeled Blue and Red. All participants signed informed consent before participating and received monetary compensation. The experiment was approved by the ethics committee of Utrecht University. For full details, see Exp. 1 in Mathôt, Van Heusden, and Van der Stigchel (2015c).

**Fig. 3:**
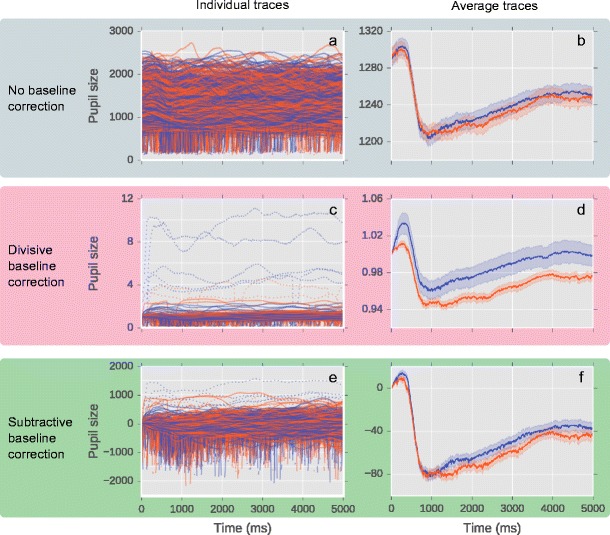
The effects of divisive and subtractive baseline correction in a real dataset. (**a**, **b**) No baseline correction. Y-axis reflects pupil size in arbitrary units. (**c**, **d**) Divisive baseline correction. Y-axis reflects proportional pupil-size change relative to baseline period. (**e**, **f**) Subtractive baseline correction. Y-axis reflects difference in pupil-size from baseline period in arbitrary units. Individual pupil traces: (**a**, **c**, **e**). Average pupil traces: (**b**, **d**, **f**)

First, consider the uncorrected data (Fig. 3a,b). The trial starts with a pronounced pupillary constriction, followed by a gradual redilation. Overall (Fig. 3b), the pupil is slightly larger in the Blue than the Red condition, but this difference is small. (Whether or not the difference between the two conditions is reliable is not relevant in this context.) The individual traces (Fig. 3a) show a lot of variability between trials, as well as frequent blinks, which correspond to the vertical spines protruding downward.

### Divisive baseline correction

Figure 3c,d shows the data after applying divisive baseline correction (applied in the same way as for the simulated data). Overall, the data now suggest that the pupil is markedly larger in the Blue than the Red condition. But to the expert eye, the pattern is odd, because the difference between Blue and Red is mostly due to a sharp (apparent) pupillary dilation in the Blue condition immediately following the baseline period; afterwards, the difference remains more-or-less constant. This is odd if you know that, because of the latency of the pupillary response, real effects on pupil size develop at the earliest about 220 ms after the manipulation that caused them (e.g., Mathôt, van der Linden, Grainger, & Vitu, 2015b); in other words, there should not be any difference between Blue and Red before 220 ms.

If we look at the individual trials, it is clear where the problem comes from: Because of blinks during the baseline period, baseline-corrected pupil size is unrealistically large in a handful of trials (dotted lines). Most of these trials are in the Blue condition, and this causes overall pupil size to be overestimated in the Blue condition. (It is not clear why there are more blinks in the Blue than the Red condition. This may well be due to chance. But even if the two conditions systematically differ in blink rate—which would be interesting—this difference should not confound the pupil-size data!)

### Subtractive baseline correction

Figure 3e,f shows the data after applying subtractive baseline correction (applied in the same way as for the simulated data). Overall, the difference in pupil size between Blue and Red is exaggerated compared to the raw data (compare Fig. 3f to b). If we look at the individual trials, this is again due to the same handful of trials (dotted lines), mostly in the Blue condition, in which pupil size is overestimated because of blinks in the baseline period. In other words, subtractive baseline correction suffers from the same problem as divisive baseline correction, but to a much lesser extent.

### Identifying problematic trials

**Fig. 4:**
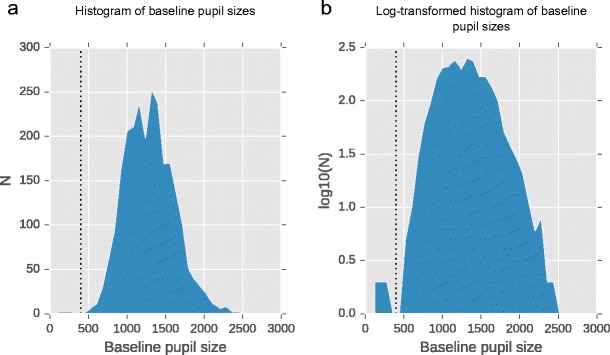
A histogram of baseline pupil sizes. (**a**) Original histogram. (**b**) Log-transformed histogram. The vertical dotted line indicates a threshold below which baseline pupil sizes appear unrealistically small

Figure 4 shows a histogram of baseline pupil sizes, that is, of median pupil sizes during the first 10 ms of the trial. In this dataset, baseline pupil sizes are more-or-less normally distributed with only a slightly elongated right tail. (But baseline pupil sizes may be distributed differently in other datasets.)

On a few trials, baseline pupil size is unusually small; but these trials are so rare that they are hardly visible in the original histogram (Fig. 4a). Therefore, we have also plotted a histogram with log-transformed counts on the Y-axis, which accentuates bins with few observations (Fig. 4b). Looking at this distribution, a reasonable cut-off seems to be 400 (arbitrary units): Baseline pupil sizes below this value are—in this dataset—unrealistic and can catastrophically affect the results as we have described above.

In Fig. 3d we have marked those trials in which baseline pupil size was less than 400 as dotted lines. As expected, those trials in which baseline-corrected pupil size is unrealistically large are exactly those trials on which baseline pupil size is unrealistically small.

**Fig. 5:**
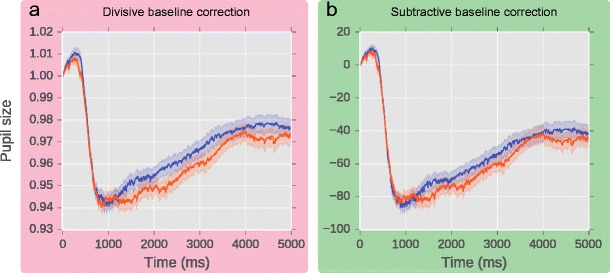
Overall results for divisive (**a**) and subtractive (**b**) baseline correction after removing trials in which baseline pupil size was less than 400

If we remove trials in which baseline pupil size was less than 400, the distortion of the overall results is much reduced. In particular, if we look at the results of the divisive baseline correction, the sharp pupillary dilation immediately following the baseline period in the Blue condition is entirely gone (compare Fig. 5a with d).

Most trials with small baselines would also have been removed if we had removed trials in which a blink was detected during the baseline. But you can think of cases in which baseline pupil size is really small while no blink is detected; for example, the eyelid can close only partly, or noisy recordings may prevent measured pupil size from going to 0 during a blink, preventing detection. Therefore, we feel that it is safer to filter based on pupil size instead of (or in addition to) filtering based on detected blinks.

## Baseline correction and statistics

Statistically speaking, what does baseline correction do?

To avoid confusion, let’s first state what baseline correction is *not*. Baseline correction is *not* a way to control for overall differences in pupil size between participants. Of course, some participants have larger pupils than others (see Tsukahara, Harrison, and Engle, 2016 for a fascinating study on the relationship between pupil size and intelligence); and the distance between camera and eye, which varies slightly from participant to participant, also affects pupil size, at least as measured by most eye trackers. But such between-subject differences are better taken into account statistically, through a repeated measures ANOVA or a linear mixed-effects model with by-participant random intercepts (e.g., Baayen, Davidson, & Bates, 2008)—just like between-subject differences in reaction times are usually taken into account.

Instead, baseline correction is a way to reduce the impact of random pupil-size fluctuations from one trial to the next. In an analysis *without* baseline correction, pupil sizes are compared between trials; for example, all trials in one condition are compared to all trials in another condition. You can think of this as a between-trial analysis. In such a between-trial analysis, random fluctuations in pupil size from one trial to the next are a source of noise, and decrease statistical power for detecting true differences between conditions.

In contrast, in an analysis *with* baseline correction, pupil sizes are first compared between the baseline epoch and another moment in the same trial. The dependent variable then becomes pupil-size change relative to baseline; pupil-size change is first determined for each trial, and then further compared between trials. You can think of this as a within-trial analysis, that is, an analysis in which Trial is taken into account as a random effect (i.e., a factor with a non-systematic effect). In a within-trial analysis, slow and random fluctuations in pupil size from one trial to the next are no longer a source of noise, and no longer decrease statistical power. This is why baseline correction improves statistical power.

**Fig. 6:**
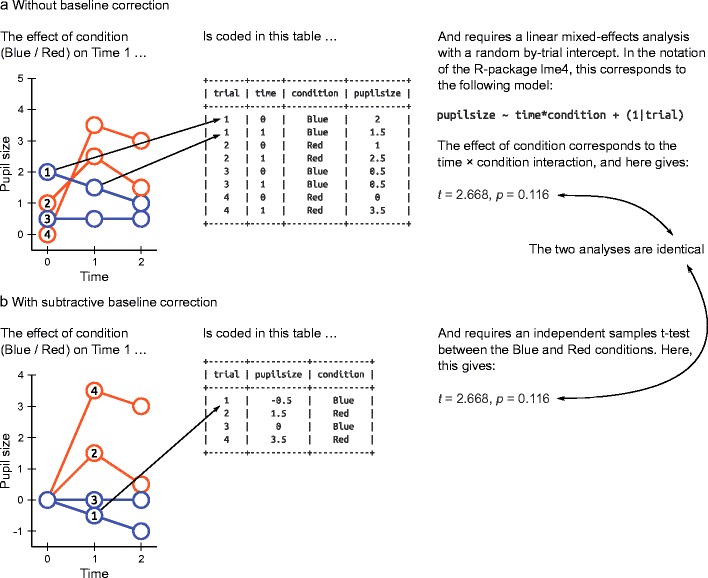
Two different ways to take baseline pupil size into account. (**a**) Baseline pupil size can be taken into account statistically, by conducting a linear mixed-effects model. (**b**) After performing subtractive baseline correction, the same analysis is reduced to independent-samples t-test

The observation that baseline correction is similar to treating Trial as a random effect deserves further consideration. Because subtractive baseline correction is not merely similar to treating Trial as a random effect—it is in every way identical. To illustrate this, let’s consider two ways to analyze pupil-size data, as shown in Fig. 6. These two ways seem very different at first sight, but are equivalent on closer inspection.

Here, we have hypothetical data of four trials with two conditions (Blue and Red). Let’s assume that we want to analyze the effect of condition at time 1. (The exact same principles would apply if we wanted to analyze the effect of condition at time 2.)

Figure 6a shows an analysis that takes baseline pupil size into account by treating Trial as a random effect, without doing explicit baseline correction. To do so, the analysis includes two time points for each trial: one time point (0) that corresponds to the baseline, and one time point (1) that corresponds to the sample that we want to analyze. We are then interested in the interaction between time and condition: This reflects the effect of condition, while taking changes in baseline pupil size into account. Because each trial now contributes two observations to the analysis, the observations in the analysis are no longer independent. To take this into account, we need to conduct a linear mixed-effects model where Trial is a random effect, and we allow the intercept to randomly vary by Trial (i.e., random by-Trial intercepts). The outcome of this analysis is *t* = 2.668, *p* = 0.116 for the time × condition interaction.

Figure 6b shows the same analysis, but done after subtractive baseline correction, so that pupil size is set to 0 at time 0 for all trials. The analysis is now reduced to a simple independent samples t-test between the Blue and Red trials. The outcome is *t* = 2.668, *p* = 0.116—identical to the linear-mixed effects analysis described above.

In other words, by treating Trial as a random effect in an analysis, you can accomplish the exact same thing as by doing subtractive baseline correction. However, in most cases baseline correction is simpler to do. Specifically, it avoids the need for complex statistical models with multiple random effects (generally at least two: Trial and Participant). This is especially relevant for researchers who prefer to analyze their data with a repeated measures ANOVA, which allows for only a single random effect, and that role is generally already reserved for Participant.

## Discussion (and five recommendations)

Here we show that baseline correction can distort pupil-size data. This happens most often when pupil size is unusually small during the baseline period, which in turn happens most often because of eye blinks, data loss, or other distortions. When baseline pupil size is unusually small, baseline-corrected pupil size becomes unusually large. This is a problem for all forms of baseline correction, but is much more pronounced for divisive than subtractive baseline correction. Therefore, subtractive baseline correction (corrected pupil size = pupil size − baseline) is more robust than divisive baseline correction (corrected pupil size = pupil size/baseline).

Despite risk of distortion, it makes sense to perform baseline correction, because it increases statistical power in experiments that investigate the effect of some experimental manipulation on pupil size. In our simulations, subtractive baseline correction increased statistical power more than divisive correction; however, we simulated a fixed difference between conditions that did not depend on baseline pupil size. For such baseline-independent effects, subtractive baseline correction leads to the highest statistical power. But real pupillary responses are always somewhat dependent on baseline pupil size, if only because a baseline pupil of 2 mm cannot constrict much further, nor can a baseline pupil of 8 mm dilate much further. The more important point is therefore that baseline correction in general increases statistical power compared to no baseline correction.

Knowing the risks and the benefits, how can you perform safe and sensible baseline correction and preprocessing of pupil-size data? Based on our observations, we make five recommendations:

1. Prior to baseline correction, perform data preprocessing data in a way that is appropriate for your research question, as described in the section *A Preprocessing Primer*. However, do not assume that preprocessing leads to perfectly clean data.
2. Use subtractive baseline correction (or some variation thereof); that is, we recommend that on the level of individual trials, baseline pupil size be subtracted from real pupil size. Other transformations can be applied as you see fit, but they should be applied to the aggregate data, and not to individual trials. For example, if you prefer to express pupil size as proportion change, you can divide pupil size by the grand mean pupil size during the baseline period averaged across all trials.
3. Visually compare your baseline-corrected data with your uncorrected data. Baseline correction should reduce variability, but not qualitatively change the overall results.
4. Baseline artifacts manifest themselves as a rapid dilation of the pupil immediately following the baseline period. Given that real effects on pupil size emerge slowly, never within 220 ms of the manipulation, baseline artifacts can be distinguished from real effects by their timing.
5. Plot a histogram of baseline pupil sizes, and use this to visually determine a minimum baseline pupil size, and remove all trials on which baseline pupil size is smaller. We do not recommend using a fixed criterion such as “remove all baseline pupil sizes that are more than 2.5 standard deviations below the mean.” While this may work in some cases (it would have worked in the real data used here), the distribution of baseline pupil sizes varies, and therefore a fixed criterion may not always catch all problematic trials. We also do not recommended relying on blink detection. Although blinks are the primary reason for unrealistically small baseline pupil sizes, they are not the only reason; furthermore, blinks may not be detected when the eyelid closes only partly or when the recording is noisy. As we’ve seen, catching all problematic trials is important, because even a handful of trials with baseline artifacts can catastrophically affect the overall results. A visually determined criterion for minimum baseline pupil size is safest.

We and many others have used baseline correction in the past, and some people, including us, have also used divisive baseline correction. In light of this paper, can we still trust these previous results? For the most part: yes. Importantly, baseline artifacts can trigger quantitatively large spurious effects, but these spurious effects are unlikely to be significant, because baseline artifacts also introduce a lot of variance. Therefore, baseline artifacts are more likely to result in false negatives (type II errors) than false positives (type I errors). We have also checked our own previous results (e.g., Blom, Mathôt, Olivers, & Van der Stigchel, 2016; Mathôt, Dalmaijer, Grainger, & Van der Stigchel, 2014) and found no signs of baseline artifacts, nor have we found obvious signs of distortion (of this kind) in other published work. Presumably, most researchers check their data visually to make sure that clear outliers are removed; therefore, catastrophic distortion (as shown in Fig. 1c,d and Fig. 3c,d) is likely to be noticed and, in one way or another, corrected. Nevertheless, although problems may be detected and dealt with in an ad hoc fashion, using a safe and sensible approach to begin with is preferable.

In conclusion, we have shown that baseline correction of pupil-size data increases statistical power, but can strongly distort data if there are artifacts (notably eye blinks) during the baseline period. We have made five recommendations for safe and sensible baseline correction, the most important of which are: Use subtractive rather than divisive baseline correction, and check visually whether your baseline-corrected pupil-size data make sense.
